# The Clinicopathological Features of BRAF Mutated Papillary Thyroid Cancers in Chinese Patients

**DOI:** 10.1155/2015/642046

**Published:** 2015-07-27

**Authors:** Li-Bo Yang, Lin-Yong Sun, Yong Jiang, Ying Tang, Zhi-Hui Li, Hong-Ying Zhang, Hong Bu, Feng Ye

**Affiliations:** ^1^Department of Pathology, West China Hospital, Sichuan University, Chengdu 610041, China; ^2^Laboratory of Pathology, West China Hospital, Sichuan University, Chengdu 610041, China; ^3^Department of Thyroid and Breast Surgery, West China Hospital, Sichuan University, Chengdu 610041, China

## Abstract

The BRAF^V600E^ mutation is commonly found in papillary thyroid cancers (PTCs) at different frequencies in different regions. However, the association between the BRAF^V600E^ mutation and clinicopathological features in Chinese PTC patients is unknown. A total of 543 Chinese patients with histologically confirmed PTC were enrolled in this study. For the BRAF mutation assay, the target fragments were amplified and sequenced with an ABI 3500 gene analyzer. In 170 of 543 samples (31.3%), the BRAF^V600E^ mutation was detected. In the bivariate analysis, the BRAF^V600E^ mutation showed an association with bilaterality, tumor size, extrathyroidal invasion, and lymph node metastases (LNM). However, in the multivariate analysis, the BRAF^V600E^ mutation was positively related to only tumor size (>1 cm) and extrathyroidal invasion. In addition, the multivariate analysis also showed that the age at diagnosis (<45 y) and tumor size (>1 cm) were independent predictors for LNM. In this study, the BRAF^V600E^ mutation is positively associated with worse prognostic factors, including larger tumor size and the tumor extending to the thyroid capsule or extrathyroidal region; however, it is not an independent predictor for LNM.

## 1. Introduction

The incidence of thyroid cancer has increased over the past few decades in different countries [1], including China [1, 2]. Among all the types of thyroid cancers, the increased rate of papillary thyroid cancer (PTC) is particularly high [3]. PTC, which is the most common subtype of thyroid cancer, represents approximately 80%–90% of all thyroid cancers [3–5].

The BRAF^V600E^ mutation leads to uncontrolled activation of the MAPK pathway, which is crucial for both tumor initiation and the progression of PTC [6, 7]. Gene mutations and/or chromosome changes in certain effectors of the MAPK pathway, such as BRAF mutations, RAS mutations, and RET/PTC rearrangements, lead to constitutive activation of cell signaling, resulting in uncontrolled proliferation and carcinogenesis. However, the association between BRAF^V600E^ mutation and clinicopathological features remains controversial. Several studies showed that the occurrence of the BRAF^V600E^ mutation in PTC was related to aggressive features, including extrathyroidal invasion and lymph node metastases [8–10]. However, other authors did not find this association [11, 12]. Lymph node metastasis (LNM) is an aggressive factor and is associated with recurrence and cancer-related mortality [13, 14]. Patients who underwent central lymph node dissection suffered a greater risk of permanent hypoparathyroidism or permanent nerve injury [15]. Preoperative medical checkups did not identify all metastases, including some small metastases in the lymph node [16]. This common clinical dilemma is troubling, although some authors reported that the BRAF^V600E^ mutation is an independent predictor of central node metastasis [17–20]. Therefore, it is necessary to determine if the BRAF^V600E^ mutation is a predictor of LNM in Chinese PTC patients and if it may help surgeons decide whether to perform lymph node dissection.

In addition, the BRAF mutation may be involved in the decreased expression of iodine metabolism genes, such as the sodium/iodide symporter and TSH receptor [21–24], leading to iodine resistance. Target inhibition of BRAF activity is currently the alternative therapeutic approach for iodine-refractory PTC [25, 26], and several small molecule inhibitors of BRAF have been developed, including selumetinib, sorafenib, BAY 43-9006, PLX4032, RAF265, and PLX4720 [26–29], each with different selectivities [30, 31]. Furthermore, some of these drugs are currently in phase II and phase III clinical trial studies for the treatment of thyroid cancer [26, 31]. By contrast, the incidence of thyroid cancer is different depending on race and geographic regions. The countries with a high reported incidence of thyroid cancer are Polynesia, Iceland, Italy, Israel, Finland, Hong Kong, China, Canada, and United States; the highest rate is found in New Caledonia, which has reported an approximately 10-fold higher rate than most developed countries [32–34]. The incidence of the BRAF^V600E^ mutation in PTC also has a wide range of frequencies, from 25 to 90% [8, 12, 35–38]. Jeong et al. [8] reported that the frequency of the BRAF mutation in Korean PTC patients was 90%. Currently, this is the highest frequency of the BRAF mutation reported. The different geographic region is the most likely explanation for this phenomenon [39]. There is a clear need to determine the frequency of the BRAF^V600E^ mutation, prior to administering any targeted clinical therapies into Chinese PTC patients.

In this study, we investigated the status of the BRAF^V600E^ mutation in primary tumors from 543 Chinese PTC patients to identify correlations between this genetic event and clinicopathological factors.

## 2. Materials and Methods

### 2.1. Patients and Samples

**Figure 1:**
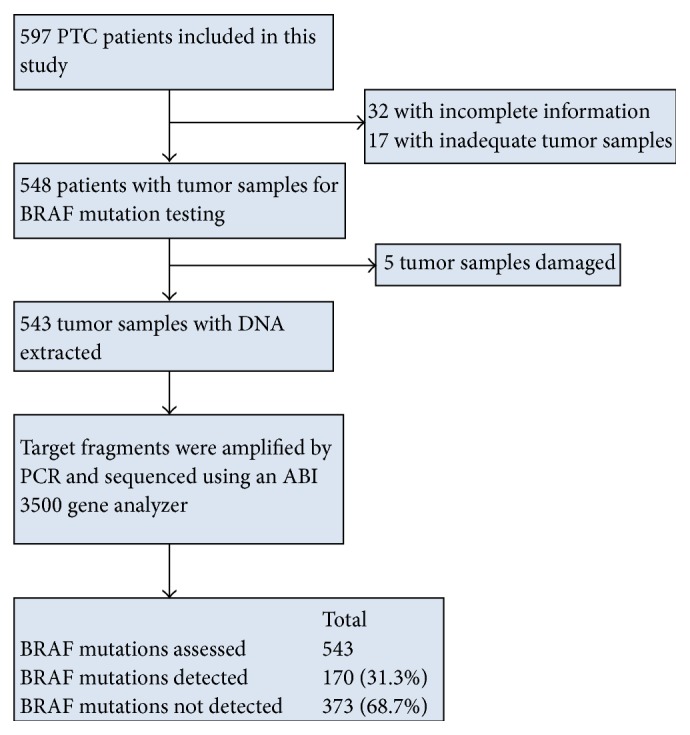
Flowchart showing the inclusion and results of the BRAF^V600E^ mutation in Chinese PTC patients.

This study was a retrospective study and all data were analyzed anonymously. The Institutional Ethics Committee of the West China Hospital approved this study. Patients with histologically confirmed PTC from January 2013 to December 2014 were assessed for this study. A total of 597 primary cases that underwent thyroidectomy and routine central lymph node dissection at the West China Hospital were selected for further study. Of these, 54 patients were excluded because of incomplete patient information or an inadequate tumor sample (Figure 1). Finally, we included 543 formalin-fixed paraffin-embedded (FFPE) PTC specimens in this study. Some patients selectively underwent lateral neck lymph node dissection if the preoperative medical checkups, such as ultrasound, computed tomography, fine needle aspiration cytology, or cervical lymph node biopsy, suggested metastatic papillary cancer. Demographic and clinicopathological features, including gender, age of the patient at diagnosis, multifocality, bilaterality, tumor size, extrathyroidal invasion, and lymph node status, were collected from the patient medical histories and pathology reports. Specifically, “extrathyroidal invasion” in this study indicates that the tumor invaded the thyroid capsule or grew into the extrathyroidal region. The International Union against Cancer/American Joint Committee on Cancer (UICC/AJCC) tumor node metastasis (TNM) classification system was used for tumor staging [40]. All information regarding these samples is presented in Table 1.

### 2.2. DNA Extraction and BRAF Analysis

A total of 2–8 sections, each with a thickness of 5 *μ*m and a surface area of approximately 200 mm^2^ in total, were used for DNA extraction. A QIAamp DNA FFPE Tissue Kit (QIAGEN cat.56404) was used for genomic DNA extraction from the FFPE blocks. Briefly, the paraffin was removed with xylene and washed with ethanol. Air-dried tissue was resuspended in ATL buffer with proteinase K and incubated overnight. After a 1 hour incubation at 90°C, 200 *μ*L of AL was added to the sample. The entire mixture was loaded onto a QIAamp MinElute column. Then, the sample was washed with AW1 and AW2 wash buffer, and the genomic DNA was eluted in 50 *μ*L of ATE buffer. The polymerase chain reaction (PCR) primers used were the forward primer, 5′-TGCTTGCTCTGATAGGAAAATG-3′, and reverse primer, 5′-AGCCTCAATTCTTACCATCCA-3′. The thermocycler program was set as follows: 94°C 3 min, 35 × (94°C 30 sec, 60°C 30 sec, and 72°C 30 sec), and 72°C for 5 min. PCR products (191 bp) were subjected to automated sequencing using an ABI PRISM 3500 (Applied Biosystems, Foster City, CA, USA). All mutated cases were confirmed twice with independent PCR assays.

### 2.3. Statistical Analysis

Statistical analysis was performed with SPSS 16.0 (SPSS, Inc., Chicago, IL). Pearson *χ* ^2^ test and Fisher's exact test were used to calculate the bivariate analysis of the relationship between the BRAF^V600E^ mutation status and clinicopathological features, including gender, age of the patient at diagnosis, multifocality, bilaterality, tumor size, extrathyroidal invasion, lymph node status, and AJCC stage. A multivariate analysis was performed using binary logistic regression analysis for variables, which were significant in the bivariate analysis. Binary logistic regression analysis was also conducted to identify the variables associated with LNM independently. These variables were assessed using preoperative examination and intraoperative frozen-section examination, including gender, age of the patient at diagnosis, multifocality, bilaterality, tumor size, and extrathyroidal invasion. Continuous variables are presented as the mean ± standard deviation. Independent samples *t*-tests were used for analysis. The two-sided significance level was set at *P* < 0.05.

## 3. Results

### 3.1. Baseline Clinicopathological Characteristics

The information for the 543 patients diagnosed with PTC included in this retrospective study is summarized in Table 1. A total of 409 females (75.3%) and 134 males (24.7%) with a mean age of 42.10 ± 12.12 years at the time of diagnosis were included. The mean tumor size was 1.14 ± 0.93 cm. Bilateral PTC was present in 125 patients (23.0%). Multifocal PTC was found in 230 patients (42.4%). A total of 359 patients (66.1%) had extrathyroidal invasion. LNM was discovered in 321 patients (59.1%). Of all PTC patients, 170 patients (31.3%) harbored BRAF^V600E^. For tumor stage, 434 patients (79.9%) were at stage I, 6 patients (1.1%) were at stage II, 70 patients (12.9%) were at stage III, and 33 patients (6.1%) were at stage IV.

### 3.2. Association between BRAF^V600E^ Mutation and Clinicopathological Characteristics

Bivariate analysis showed that the tumor size, bilaterality, extrathyroidal invasion and LNM are associated with BRAF^V600E^ (Table 2). There was no significant association of the BRAF^V600E^ mutation with gender, age at diagnosis, multifocality, and AJCC stage in our study. The relationship between BRAF^V600E^ and tumor size were confirmed by *t*-test. The mean tumor size of the 170 patients with BRAF^V600E^ was 1.47 ± 0.99 cm, whereas the mean tumor size of the wild type patients was 0.98 ± 0.87 cm (*t* = 5.80, *P* < 0.001).

Additionally, multivariate analysis (binary logistic regression) showed that BRAF^V600E^ was positively associated only with tumor size (>1 cm) and extrathyroidal invasion (Table 3).

### 3.3. Predictive Factors of LNM

We then performed a binary logistic regression analysis to determine whether BRAF^V600E^ is an independent predictive factor for central LNM (Table 4) or lateral LNM (Table 5). After controlling for gender, age at diagnosis, multifocality, bilaterality, extrathyroidal invasion, tumor size, and BRAF^V600E^ mutation, we found that the BRAF^V600E^ mutation was not an independent predictor for central LNM or lateral LNM. However, age at diagnosis (<45 y) and tumor size (>1 cm) were independent predictors for both central LNM and lateral LNM. Gender (female) was an independent predictor for only central LNM, whereas bilateral tumor and extrathyroidal invasion were independent predictors for only lateral LNM.

## 4. Discussion

Sanger sequencing, PCR, and immunohistochemistry are three primary methods for detecting the BRAF mutation. There were no significant differences among these methods [41]. However, PCR is commonly used because of its high-efficiency; therefore, we only used this method to detect mutations in this study. There are more than ten types of BRAF mutation variants reported for malignant tumors such as bladder, melanoma, and PTC. These variants include BRAF^V600E^, BRAF^V600D^, BRAF^V600M^, BRAF^V600V^, BRAF^V600R^, BRAF^V600E2^, BRAF^V600Q^, BRAF^V600L^, and BRAF^V600K^ [42–44]. However, in our study, we only identified the common type of BRAF mutation variant, BRAF^V600E^. Therefore, theoretically, any drugs that specifically inhibit B-type RAF kinase should at least target the mutant type to inhibit the MAPK signaling pathway. Several studies reported probability of using a BRAF mutation inhibitor in iodine-refractory PTC [25, 26]. This requires additional large-scale prospective trail to determine the availability and safety in patients.

The frequency of BRAF^V600E^ in Chinese PTC patients is 31.3% (170/543). The rate for BRAF^V600E^ mutation is approximately the same as reported in other countries [6, 7, 31]. We did not identify any other BRAF mutation variant types as described in other studies. This may occur in a race-dependent manner. Furthermore, the sample size was not large enough to identify all reported BRAF genetic changes.

Additionally, we analyzed the BRAF^V600E^ mutation and its clinical and pathological characteristics. Some studies reported that the BRAF^V600E^ mutation is related to male gender, older age, tumor size, thyroid capsular invasion, extrathyroidal extension, and LNM (Table 6) [18, 45–49]. These features indicated a poor outcome in PTC patients [50–52]. In our study, the BRAF^V600E^ mutation had a relationship with the tumor size, bilaterality, extrathyroidal invasion, and LNM based on the bivariate analysis. Multivariate analysis showed that tumor size (>1 cm) and extrathyroidal invasion had significant positive associations with the BRAF^V600E^ mutation, after controlling for the tumor size, bilaterality, extrathyroidal invasion, and LNM. This indicates that patients with a larger tumor size (>1 cm) or tumors extending to the thyroid capsule and extrathyroidal region are more likely to have BRAF^V600E^ mutation. In this study, we regard thyroid capsular invasion and extrathyroidal extension as “extrathyroidal invasion” to reduce errors, because the manner by which to distinguish extrathyroidal extension remains controversial. Although the publishers of the UICC/AJCC 7th edition TNM classification system have taken the degree of extrathyroidal extension into consideration, the criteria for defining extrathyroid extension are subjective and problematic because of the discontinuous capsule of the thyroid gland [53].

Several studies further defined the relationship between the BRAF mutation and the aggressiveness of thyroid tumor cells. Epithelial mesenchymal transition (EMT) is common in PTC invasion and is associated with LNM [54]. The BRAF mutation may render thyroid cells susceptible to transforming growth factor beta-induced EMT [55]. The aberrant methylation of tumor suppressor genes, leading to the increased aggressiveness of thyroid tumor cells, is also related to the BRAF mutation [56]. These studies help us understand the importance of this type of mutation in LNM. Several authors determined whether the BRAF^V600E^ mutation is a predictive factor for LNM to help surgeons decide which PTC patients should have lymph node dissection [17–19]. In our study, we did not find that the BRAF^V600E^ mutation was an independent predictor for LNM in Chinese PTC patients. However, the binary logistic regression analysis revealed that being female, younger age at diagnosis (<45 y), and tumor size >1 cm had a more significant association with central LNM. For lateral LNM, younger age at diagnosis (<45 y), bilateral tumor, extrathyroidal invasion, and larger tumor size (>1 cm) were related. Therefore, Chinese PTC patients who are young (<45 y) and have a larger tumor size (>1 cm) tend to have both central LNM and lateral LNM.

## 5. Conclusion

Our study shows that the occurrence of the BRAF^V600E^ mutation in Chinese patients is approximately the same as the other countries. The BRAF^V600E^ mutation is positively associated with worse prognostic factors, including larger tumor size and tumors extending to the thyroid capsule or extrathyroidal region. However, in our study, the BRAF^V600E^ mutation does not show predictive value for LNM.

## Figures and Tables

**Table 1:** Clinicopathological features in the study of PTC in this study.

Characteristics	Number
Number of patients	543
Gender	
Female	409 (75.3%)
Male	134 (24.7%)
Age at diagnosis, years	
Mean ± SD	42.10 ± 12.12
Range	
<45	219 (40.3%)
≥45	324 (59.7%)
Multifocality	230 (42.4%)
Bilaterality	125 (23.0%)
Tumor size, cm	
Mean ± SD	1.14 ± 0.93
Range	
≤1	345 (63.5%)
(1,2]	147 (27.1%)
(2,4]	42 (7.7%)
>4	9 (1.7%)
Extra-thyroidal invasion	359 (66.1%)
LNM	321 (59.1%)
Central LNM	312 (57.5%)
Lateral LNM	123 (22.7%)
BRAF^V600E^ mutation	170 (31.3%)
AJCC stage	
I	434 (79.9%)
II	6 (1.1%)
III	70 (12.9%)
IV	33 (6.1%)

**Table 2:** Relationship between the BRAF^V600E^ mutation and clinicopathological factors in PTC.

	Total, *n* (%)	BRAF^V600E^ mutation, *n* (%)		*χ* ^2^	*P* value
		Mutation (*n* = 170)	Wild (*n* = 373)		
Gender				2.288	0.130
Female	409 (75.3)	121 (29.6)	288 (70.4)		
Male	134 (24.7)	49 (36.6)	85 (63.4)		
Age at diagnosis				0.234	0.629
<45	219 (40.3)	66 (30.1)	153 (69.9)		
≥45	324 (59.7)	104 (32.1)	220 (69.7)		
Multifocality				0.314	0.575
No	313 (57.6)	95 (30.4)	218 (69.6)		
Yes	230 (42.4)	75 (32.6)	155 (67.4)		
Bilaterality				4.703	0.030
No	418 (77.0)	121 (28.9)	297 (71.1)		
Yes	125 (23.0)	49 (39.2)	76 (60.8)		
Tumor size (cm)				31.109	<0.001
≤1	345 (63.5)	79 (22.9)	266 (77.1)		
>1	198 (36.5)	91 (46.0)	107 (54.0)		
Extrathyroidal invasion				23.142	<0.001
No	184 (33.9)	33 (17.9)	151 (82.1)		
Yes	359 (66.1)	137 (38.2)	222 (61.8)		
LNM				7.452	0.006
No	222 (40.9)	55 (24.8)	167 (75.2)		
Yes	321 (59.1)	115 (35.8)	206 (64.2)		
AJCC stage				6.741	0.072
I	434 (79.9)	132 (30.4)	302 (69.6)		
II	6 (1.1)	2 (33.3)	4 (66.7)		
III	70 (12.9)	19 (27.1)	51 (72.9)		
IV	33 (6.1)	17 (51.5)	16 (48.5)		

**Table 3:** Multivariate analysis of the association between clinicopathological features and BRAF^V600E^ mutation.

Features	Odds ratio	95% confidence interval		*P* value
		Lower bound	Upper bound	
Bilaterality (+)	0.843	0.541	1.314	0.451
Extrathyroidal invasion (+)	2.284	1.458	3.576	<0.001
Tumor size (>1 cm)	2.319	1.548	3.473	<0.001
LNM (+)	1.172	0.774	1.774	0.454

**Table 4:** Multivariate analysis of the association between clinicopathological features and central LNM.

Features	Odds ratio	95% confidence interval		*P* value
		Lower bound	Upper bound	
Gender (female)	2.441	1.552	3.840	<0.001
Age at diagnosis (<45 y)	2.661	1.818	3.894	<0.001
Multifocality (+)	1.115	0.692	1.797	0.655
Bilaterality (+)	1.641	0.911	2.957	0.099
Extrathyroidal invasion (+)	1.389	0.932	2.072	0.107
Tumor size (>1 cm)	3.288	2.157	5.010	<0.001
BRAF^V600E^ mutation (+)	0.954	0.624	1.457	0.826

**Table 5:** Multivariate analysis of the association between clinicopathological features and lateral LNM.

Features	Odds ratio	95% confidence interval		*P* value
		Lower bound	Upper bound	
Gender (female)	1.677	0.991	2.840	0.054
Age at diagnosis (<45 y)	2.625	1.582	4.356	<0.001
Multifocality (+)	1.240	0.652	2.361	0.512
Bilaterality (+)	2.343	1.180	4.653	0.015
Extrathyroidal invasion (+)	2.323	1.304	4.136	0.004
Tumor size (>1 cm)	4.821	2.997	7.755	<0.001
BRAF^V600E^ mutation (+)	1.175	0.722	1.911	0.516

**Table 6:** Clinicopathological features associated with BRAF mutation in other studies.

Studies	Country	Numbers of PTC patients	Clinicopathological features associated with BRAF mutation
Howell et al. [18]	USA	156	Central LNM
Lim et al. [45]	Korea	3130	Tumor size, extrathyroidal extension, and LNM
Kim et al. [46]	Korea	547	Male gender, tumor size, and extrathyroidal extension
Xing et al. [47]	USA	190	Extrathyroidal extension, thyroid capsule invasion, and LNM
Nakayama et al. [48]	Japan	54	Older age, extrathyroidal extension, and LNM
Fugazzola et al. [49]	Italy	260	Older age
